# Do flickering LED lights reduce productivity of layer pullets and hens?

**DOI:** 10.1016/j.psj.2024.103456

**Published:** 2024-01-11

**Authors:** S. McPhee, T. Shynkaruk, K. Buchynski, D. Beaulieu, J. Brown, T. Crowe, K. Schwean-Lardner

**Affiliations:** ⁎ Department of Animal and Poultry Science, University of Saskatchewan, Saskatoon, Saskatchewan, Canada, S7N5A8; † Department of Mechanical Engineering, University of Saskatchewan, Saskatoon, Saskatchewan, Canada, S7N5A9

**Keywords:** Lohmann Brown-Lite, Lohmann LSL-Lite, egg production, flicker frequency

## Abstract

Most characteristics of artificial light sources are well studied, however light-flicker frequency (**F**) has been overlooked. The purpose of this study was to determine the effect of F on performance of Lohmann LSL-Lite (**LW**) pullets and Lohmann Brown-Lite (**LB**) pullets. In addition, pullets were followed through to the laying phase to evaluate long-term effects of F during rearing on productivity. Two trials were conducted with 3 F (30, 90, or 250 Hz) treatments. LW and LB pullets (*n* = 2,688 per strain [**S**]) were randomly assigned to floor pens within 8 light-tight rooms (15 pen replicates per F × S for 30 and 250 Hz; 18 pen replicates per F × S for 90 Hz). At 16 wk, pullets were transferred to conventional layer cages, with no flicker treatment applied. Pullet data collected included BW, feed disappearance, flock uniformity, and overall mortality. Hen data collected included BW, feed intake (feed efficiency calculated), mortality, egg production, and egg quality. Data were analyzed using Proc Mixed (SAS 9.4) and differences were considered significant when *P* ≤ 0.05. Frequency did not affect pullet uniformity or feed disappearance (0–8 wk and 0–16 wk). Pullets reared under 30 Hz had higher mortality (caused by “other”) than those reared under 250 Hz. Lohmann Brown-Lite pullets reared under 30 Hz had the highest feed disappearance. Overall mortality was higher for LW pullets reared under 30 Hz compared to LB reared under 30 Hz or 250 Hz. Lohmann Brown-Lite hens reared under 30 Hz were heavier at the beginning of the hen phase (17 wk), however differences related to F were not seen at 40 or 48 wk. Hen day production (%) was higher for hens reared under 30 compared to 90 Hz (*P* = 0.03), however no other egg parameters were affected by F. Hen feed efficiency and mortality were unaffected by F. These results indicate minor effects of F, during either the pullet or hen phases. The data also suggest that S (LW vs. LB) may affect response to F.

## INTRODUCTION

Light is essential for poultry production and many commercial poultry houses rely on artificial light to meet the needs of their flock. Incandescent, fluorescent, and light-emitting diode (**LED**) lamps are some options available to producers, with LED lamps gaining popularity due to their energy efficiency, long operating life, and potential to emit different wavelengths (Huber-Eicher et al., 2013; Parvin et al., 2014). One downfall to using artificial sources of light is that those powered by alternate current will flicker (IEEE, 2015). Light flicker is defined as rapid, repeated changes to the light intensity often caused by changes to current supplied to that light source (Brundrett, 1974; Prescott et al., 2003; IEEE, 2015).

While most light sources are flickering, it may not be perceived by an observer. The point when flickering light is no longer perceived as flickering and is instead seen as a continuous stream of light is called the flicker fusion frequency (**FFF**; Brundrett, 1974; Lisney et al., 2012). The highest FFF regardless of light intensity is known as the critical flicker fusion frequency (**CFF**) (Lisney et al., 2012; IEEE, 2015). In laying hens, the FFF has been determined to range from 90 to 105 Hz (Nuboer et al., 1992; Jarvis et al., 2002; Railton et al., 2009; Lisney et al., 2012). While conscious perception may not exceed 105 Hz, the retina of hens responded to flicker up to 119 Hz, indicating a level of unconscious perception (Lisney et al., 2012). This has been termed “invisible flicker” or flicker that occurs but is not consciously perceived, which may still have physiological impacts on the observer (IEEE, 2015).

The effects of light-flicker frequency (**F**) from fluorescent lamps on the behavior and stress response of European starlings has been evaluated (Maddocks et al., 2001; Greenwood et al., 2004; Smith et al., 2005; Evans et al., 2006; Evans et al., 2012). Only a few studies have evaluated the impacts of F on domestic poultry species (Boshouwers and Nicaise, 1992; Widowski and Duncan, 1996; Kavtarashvili and Gladin, 2022; Raabe et al., 2023). In broilers, a lower F of 100 Hz decreased activity, although no effect was observed on energy expenditure of the birds compared to a higher F of 26,000 Hz (Boshouwers and Nicaise, 1992). Laying hens did not show a preference for either high- (20,000–60,000 Hz) or low-frequency fluorescent flicker (120 Hz) and all behaviors examined were performed equally under both treatments (Widowski and Duncan, 1996). In terms of the effects of F on productivity of domestic species, effects have been examined in laying hens and turkeys (Kavtarashvili and Gladin, 2022; Raabe et al., 2023). Laying hens experienced higher mortality due to aggression and cannibalism, higher final body weight (BW) and higher feed consumption when housed under 120 Hz compared to a higher F (Kavtarashvili and Gladin, 2022). Egg production was lowest in hens housed under 120 Hz but they produced heavier eggs (Kavtarashvili and Gladin, 2022). Overall, Kavtarashvili and Gladin (2022) concluded that F of 120 Hz has a negative impact on hen livability and production compared to higher F. In turkey toms, final BW, overall feed consumption, injuries and mortalities did not differ when reared under a F of 165 Hz, 500 Hz, or 16 kHz (Raabe et al., 2023). The authors suggested that frequencies of 165 Hz and above have no detrimental effects on production and injurious pecking of turkey toms (Raabe et al., 2023).

Currently in Canada, there are no requirements or recommendations regarding minimum F in pullet houses (National Farm Animal Care Council, 2017), likely due to the lack of research in this area. To the author's knowledge, there is no research on the effect of light flicker on pullet production and health or the potential carryover effects of exposure to light flicker during the rearing phase on subsequent laying hen performance. It has been suggested that high stress levels in laying hen stock can have a negative impact on productivity traits (de Haas et al., 2013). Therefore, this study aimed to determine the effect of F (30 [visible to humans and birds], 90 (potentially visible to some birds but not to humans), or 250 Hz (not visible to humans or birds)) on BW, flock uniformity, feed disappearance, and mortality of 2 commercially available strains of layer pullets. Pullets were then followed through the production period to evaluate carryover effects on hen BW, feed intake, feed efficiency, mortality, egg production, and egg quality.

## MATERIALS AND METHODS

The University of Saskatchewan Animal Care Committee approved the experimental procedures for this research. The Canadian Council of Animal Care's Guide to the Care and Use of Experimental Animals (2009) was followed for the treatment and care of all birds. The primary objective of this manuscript focused on the effects of three frequencies of light flicker on the BW, uniformity, feed disappearance, and mortality of 2 strains: Lohmann LSL-Lite **(LW)** pullets and Lohmann Brown-Lite **(LB)** pullets. The secondary objective was to evaluate the carryover effect of light flicker during the rearing phase on laying hen (LW and LB) performance when housed under typical lighting in North America (120 Hz – double the AC power). The pullet portion of this experiment took place over two 16-wk trials, followed by the hen phase (17–48 wk).

### Housing and Management

A total of 1,344 of each LB and LW pullets per trial were housed in floor pens within light tight, individually controlled rooms from day of hatch to 16 wk. Each room contained 6 pens (4.0 m x 2.3 m) and pens housed 56 pullets each. The pens were bedded with wood shavings 7 to 10 cm deep and contained a wooden perching system (height 0.56 m x width 1.16 m x length 2.18 m), 2 tube feeders with pans (0.36 m diameter [0–8 wk] and 0.44 m diameter [8-16 wk]), and 1 drinker line with 6 nipples (Lubing Systems LP, Cleveland, TN, USA). During the first week, pullets had access to supplemental feeders and drinkers. Pullets had ad libitum access to a 5-phase commercial feed program and water throughout the trial. At the hatchery, all pullets were vaccinated for Marek's Rispens, HVT-IBD, and Poulvac ST. Pullets were also vaccinated for Newcastle Bronchitis, *Salmonella enteritidis*, and *Salmonella typhimurium* at various times throughout the rearing period as per vaccine requirements. Breeder flock ages for the first trial were 31 wk (LB and LW) and 32 (LB) and 53 wk (LW) for the second trial.

Rooms were randomly assigned to 1 of 3 F treatments (30, 90, 250 Hz). Light was provided with ten 11-Watt white LED lamps (AgriLamp 11W ES26/27; Greengage Lighting Ltd., Edinburgh, UK) in each room. Purpose-built flicker electronics (Greengage Lighting Ltd., Edinburgh, UK) were used to control F. Every 2 wk, F was confirmed in each room using either a spectrometer (Lighting Passport Spectrometer, ASENSETEK, Xindian District, Taiwan) or a Lichtflimmer (**LiFli**) (Messgerat LiFli, Fauser Elektrotechnik, München, Germany) with an oscilloscope (TDS 210 Digital Real-Time Oscilloscope, Tektronix, Beaverton, OR, USA) to ensure experimental conditions were maintained. For the first 7 d, pullets were raised under 22L:2D before decreasing abruptly to 8L:16D for the remainder of the trial. Light intensity throughout the trial was maintained at 30 lux and was confirmed every 2 wk using a luxmeter (ExTech LT300; ExTech Instruments, Montreal, Canada). Room temperature at placement was 33°C during the first wk and was gradually decreased to 20°C by 5 wk, where it was maintained for the duration of the trial.

At 16 wk, 864 pullets were transferred to the layer barn, where they were housed in conventional cages (60 × 50 × 40 cm). A total of 36 replicates for each strain (LB and LW) were used, with the replicate unit being 2 cages (6 hens/cage), which share a single feed trough. Each cage was equipped with 4 nipple drinkers. The hens were fed commercial layer diets, appropriate for the current phase of egg production. A photoperiod of 8L:16D was used from 16 to 18 wk. At 19 wk, photoperiod was increased to 12L:12D and an additional hour of light was added weekly to reach 16L:8D at 23 wk. The light in the hen barn was provided via incandescent bulbs. Light intensity was 10 lux and flicker frequency was 120 Hz (not within the visible range for hens).

### Data Collection

*Pullet Body Weight and Uniformity****.*** Pullets were weighed on a pen basis at 0, 8, and 16 wk and average BW per bird was calculated. Uniformity was assessed at 16 wk by individually weighing 288 random pullets per F treatment (*n* = 96 pens).

*Pullet Feed Disappearance.* Feed was weighed into each pen and remaining feed was weighed at 8 and 16 wk to calculate feed disappearance for each period (kg per bird per day) (*n* = 96 pens).

*Pullet Mortality.* Pullets were checked twice daily for mortality and culls throughout the trial. A Humane Intervention Point Checklist (University of Saskatchewan Poultry Centre SOP Guide, 2021) was used to determine if euthanasia was necessary (*n* = 96 pens). All mortality was submitted to an independent diagnostic laboratory (Prairie Diagnostic Services, Saskatoon, SK, Canada) for necropsy and cause of morbidity or mortality was determined.

*Hen Performance.* Hens were weighed individually at 17, 40, and 48 wk. Feed intake was measured on a replicate basis (2 cages) every 4 wk and feed efficiency was calculated. Mortality and culls were recorded daily and were submitted for diagnosis as described above. Egg production was recorded 5 d per week throughout the production cycle and included: number of eggs laid and number of double yolks, cracked, broken, soft, and abnormal eggs. Egg quality was evaluated every 8 wk and included: egg weight and specific gravity on all eggs laid that day as well as shell thickness and albumen height on 6 eggs/replicate.

### Statistical Analyses

The experiment utilized a randomized complete block design (trial as block) with room nested within F. Treatments were arranged as a 3 (F) x 2 (S) factorial arrangement. The significance of the block was tested and removed from the analysis when not significant. Percentage data were checked for normality using the Proc Univariate in SAS 9.4 (Cary, NC) and normalized using log transformation (log +1) where necessary. Data were analyzed using Proc Mixed (SAS 9.4, Cary, NC) with room as the replicate unit for F (5 replications for 30 Hz and 250 Hz; 6 replications for 90 Hz) and pen as the replicate unit for S (3 replicates per S per room per trial). A Tukey's range test was used to separate means. Significance was declared when *P* ≤ 0.05.

## RESULTS

### Pullet Body Weight and Uniformity

At d 0, LW pullets were heavier than LB pullets (*P* < 0.01; Table 1). Interactions were noted between F and S at 8 and 16 wk as LB weighed more than LW pullets regardless of F (*P* = 0.05 and *P* < 0.01, respectively; Table 1). Light-flicker frequency did not affect pullet uniformity, but LW had improved uniformity, with a higher percentage of pullets within 5, 10, and 15% of the mean, as well as having a lower coefficient of variation (*P* < 0.01; Table 2).

**Table 1:** Average pullet body weight (kg) per bird of 2 strains of pullets (S; Lohmann Brown-Lite [LB] and Lohmann LSL-Lite [LW]) reared in floor pens under light-flicker frequency (F) of 30, 90, or 250 Hz.

Age (wk)	F				*P*-value		S		*P-*value		F x S *P-*value		SEM^1^
	30	90		250			LB	LW					
0	0.036	0.036		0.035	0.94		0.035^b^	0.037^a^	<0.01		0.89		0.0002
8^2^	0.712	0.708		0.693	0.17		0.723^a^	0.686^b^	<0.01		0.05		0.0027
16^2^	1.307	1.304		1.293	0.36		1.407^a^	1.196^b^	<0.01		<0.01		0.0111

*F x S interaction – body weight at 8 and 16 wk*													
	LB							LW					
Age (wk)	30		90			250		30		90		250	

8	0.735^a^		0.727^a^			0.706^ab^		0.690^bc^		0.689^bc^		0.679^c^	
16	1.424^a^		1.403^a^			1.395^a^		1.190^b^		1.205^b^		1.192^b^	

a,b,c Means within a main effect and within an interaction with different superscripts are significantly different (*P* ≤ 0.05).

1 SEM: standard error of the mean.

2 Block was not significant and was removed from analysis.

**Table 2:** Body weight uniformity of 2 strains (S; Lohmann Brown-Lite [LB] and Lohmann LSL-Lite [LW]) of pullets reared in floor pens under light-flicker frequency (F) of 30, 90, or 250 Hz at 16 wk of age.

% within x of the mean	F			*P-*value	S		*P-*value	F x S *P-*value	SEM^1^
	30	90	250		LB	LW			
5%^2^	58.47	61.15	60.69	0.54	54.20^b^	66.24^a^	<0.01	0.50	1.591
10%^2^	89.50	91.35	90.83	0.69	86.61^b^	94.67^a^	<0.01	0.26	0.841
15%^2^	98.47	98.33	98.54	0.95	97.27^b^	99.63^a^	<0.01	0.77	0.305
CV^2^	6.13	6.07	5.99	0.92	6.94^a^	5.19^b^	<0.01	0.71	0.153

a,b Means within a main effect with different superscripts are significantly different (*P* ≤ 0.05).

1 SEM: standard error of the mean.

2 Block was not significant and was removed from analysis.

### Pullet Feed Disappearance

Light-flicker frequency had no impact on feed disappearance during the 0 to 8 or 0 to 16 wk periods (Table 3). However, during both of those periods, LB pullets had higher feed disappearance than LW (*P* < 0.01; Table 3). During the 8 to 16 wk period, an interaction was noted between F and S indicating that LB pullets reared under 30 Hz had the highest feed disappearance, followed by LB pullets reared under 90 and 250 Hz, then all LW pullet treatments (*P* < 0.01; Table 3).

**Table 3:** Average feed disappearance (kg/bird/day) for 2 strains (S; Lohmann Brown-Lite [LB] and Lohmann LSL-Lite [LW]) of pullets reared in floor pens under light-flicker frequency (F) of 30, 90, or 250 Hz.

Period (wk)	F				*P-*value		S		*P-*value		F x S *P-*value	SEM^1^
	30	90		250			LB	LW				
0-8	0.040	0.040		0.036	0.30		0.042^a^	0.037^b^	<0.01		0.79	0.0006
8-16	0.060^a^	0.059^b^		0.058^b^	0.01		0.061^a^	0.057^b^	<0.01		<0.01	0.0003
0-16	0.050	0.050		0.050	0.15		0.052^a^	0.047^b^	<0.01		0.48	0.0003

*F x S interaction – feed disappearance 8-16 wk*												
			F									
S			30			90				250		

LB			0.063^a^			0.060^b^				0.060^b^		
LW			0.057^c^			0.057^c^				0.057^c^		

a,b,c Means within a main effect and within an interaction with different superscripts are significantly different (*P* ≤ 0.05).

1 SEM: standard error of the mean.

### Pullet Mortality

Pullets reared under 30 Hz had more diagnoses in the “other” category of mortality than those reared under 250 Hz (*P* = 0.02; Table 4). The LW pullets had more infectious diagnoses as a cause of mortality, specifically yolk sac infections, than LB pullets (*P* < 0.01; Table 4). An interaction between F and S was noted for overall mortality indicating that LW pullets reared under 30 Hz had a higher overall mortality than LB pullets reared under 30 Hz or 250 Hz (*P* = 0.03; Table 4). Strains reacted differently to F with respect to level of mortality due to dehydration, despite overall levels being very low (0–0.60%). Mortality of LW pullets reared under 250 Hz were diagnosed more often as due to dehydration than all other treatments, except for LB pullets reared under 90 Hz (*P* < 0.01; Table 4).

**Table 4:** Overall mortality (% of birds placed) and mortality by cause of 2 strains (S; Lohmann Brown-Lite [LB] and Lohmann LSL-Lite [LW]) of pullets reared in floor pens under light-flicker frequency (F) of 30, 90, or 250 Hz from 0 to 16 wk of age.

Cause	F				*P*-value	S			*P*-value		F x S *P*-value		SEM^1^
	30	90		250		LB		LW					
Overall mortality	1.71	1.34		1.29	0.79	0.97^b^		1.86^a^	<0.01		0.03		0.159
Infectious	0.60	0.55		0.50	0.81	0.11^b^		0.97^a^	<0.01		0.92		0.103
Yolk sac infection	0.52	0.50		0.35	0.46	0.04^b^		0.86^a^	<0.01		0.59		0.099
Peritonitis	0.07	0.50		0.15	0.60	0.07		0.11	0.55		0.43		0.041
Skeletal	0	0		0.05	0.51	0.04		0	0.39		0.44		0.019
Emaciation/Dehydration	0.15	0.35		0.40	0.40	0.37		0.26	0.40		<0.01		0.075
Emaciation	0.15	0.15		0.10	0.90	0.22		0.04	0.08		0.46		0.048
Dehydration	0	0.20		0.30	0.11	0.15		0.22	0.63		<0.01		0.062
“Other”/NVL^2^	0.97^a^	0.45^ab^		0.35^b^	0.03	0.45		0.63	0.15		0.08		0.099
“Other”^3^	0.37^a^	0.20^ab^		0^b^	0.02	0.11		0.22	0.16		0.18		0.053
NVL^2^	0.60	0.25		0.35	0.28	0.34		0.41	0.49		0.16		0.083

*F x S interactions – mortality*													

	LB						LW						
	30		90		250		30			90		250	

Overall mortality	0.89^bc^		1.39abc		0.60^c^		2.53^a^			1.29abc		1.98^ab^	
Dehydration	0^b^		0.40^ab^		0^b^		0^b^			0^b^		0.60^a^	

a,b,c Means within a main effect and within an interaction with different superscripts are significantly different (*P* ≤ 0.05).

1 SEM: standard error of the mean.

2 NVL: no visible lesions.

3 “Other” includes prolapse, choristoma, presence of urates in ureter, and undefined.

### Hen Performance

Hen body weight at 17 wk demonstrated a FxS interaction with LB hens reared under 30 Hz being heavier than those reared under 90 and 250 Hz, followed by all LW hens (*P* = 0.01; Table 5). There was no impact of F on hen BW at 40 or 48 wk of age. At both 40 and 48 wk, S was significant with LB hens being heavier than LW hens (*P* < 0.01; Table 5). There were no FxS interactions for feed intake or feed efficiency. Feed intake was not impacted by F in the pullet rearing phase; however, LB hens consumed more feed than LW hens (*P* < 0.01; Table 6). Feed efficiency (feed consumed per egg mass and feed consumed per dozen eggs) was unaffected by F treatment. Feed consumed per egg mass was unaffected by S, while feed consumed per dozen eggs was found to be higher for the LW hens compared with the LB (*P* = 0.01; Table 6). There were no interactions between F and S for egg production and quality. Egg production (hen day production [%]) was higher in hens reared under 30 Hz compared to 90 Hz, with 250 Hz being intermediate (*P* = 0.03; Table 7). Flicker treatment did not affect any of the other egg production or quality parameters (Table 7). There were multiple S differences found, with LB hens having higher hen day and hen housed production than LW (*P* < 0.01; Table 7). The LW hens had improved egg quality compared with LB, with fewer double yolk, soft shell, cracked, abnormal eggs, or total unsalable eggs (*P* < 0.01; Table 7). The LW hens also had heavier egg weights, higher specific gravity and higher albumen height measurements compared with LB (*P* < 0.01; Table 7). Finally, hen mortality demonstrated no interactions for F and S, as well as no effect of F treatment (Table 8). Mortality demonstrated a few S differences, with LB hens having higher total mortality, as well as increased mortality due to cannibalism and mechanical injuries (broken bones) compared with LW hens (*P* < 0.01; Table 8).

**Table 5:** Impact of light-flicker frequency (F; 30, 90, or 250 Hz) during the pullet rearing phase on the body weight (kg) of 2 strains (S; Lohmann Brown-Lite [LB] and Lohmann LSL-Lite [LW]) of hens at 16, 40, and 48 wk of age.

Age (wk)	F			*P*-value		S		*P*-value		F x S *P*-value	SEM^1^
	30	90	250			LB	LW				
17	1.32^a^	1.31^ab^	1.30^b^	0.04		1.42^a^	1.20^b^	<0.01		0.01	0.009
40	1.92	1.92	1.91	0.71		2.03^a^	1.81^b^	<0.01		0.70	0.011
48	1.96	1.96	1.95	0.86		2.05^a^	1.86^b^	<0.01		0.48	0.010

*F x S interaction – hen body weight (kg)*											

	F										
S	30				90				250		

LB	1.43^a^				1.41^b^				1.40^b^		
LW	1.20^c^				1.21^c^				1.20^c^		

a,b,c Means within a main effect and within an interaction with different superscripts are significantly different (*P* ≤ 0.05).

1 SEM: standard error of the mean.

**Table 6:** Effect of light-flicker frequency (F; 30, 90, or 250 Hz) during the pullet rearing phase on the feed intake (g/bird/day) and feed efficiency of 2 strains (S; Lohmann Brown-Lite (LB) and Lohmann LSL-Lite (LW)) of hens housed in conventional cages from 17 to 48 wk of age.

	F			*P*-value	S		*P*-value	F x S *P*-value	SEM^1^
	30	90	250		LB	LW			
Feed intake	105.98	105.71	105.58	0.77	107.09^a^	104.42^b^	<0.01	0.94	0.269
TFEM^2^	2.14	2.15	2.13	0.94	2.13	2.15	0.40	0.93	0.016
TFDE^3^	1.52	1.52	1.51	0.92	1.49^b^	1.54^a^	0.01	0.85	0.009

a,b Means within a main effect with different superscripts are significantly different (*P* ≤ 0.05).

1 SEM: standard error of the mean.

2 TFEM: total feed per egg mass.

3 TFDE: total feed per dozen eggs.

**Table 7:** Effect of light-flicker frequency (F; 30, 90, or 250 Hz) during the pullet rearing phase on egg production and quality parameters for 2 strains (S; Lohmann Brown-Lite [LB] and Lohmann LSL-Lite [LW]) of hens housed in conventional cages from 17 to 48 wk of age.

Parameter	F					*P*-value	S		*P*-value	F x S *P*-value	SEM^1^
	30	90		250			LB	LW			
Hen day production, %	87.02^a^	86.84^b^		86.88^ab^		0.03	88.24^a^	85.58^b^	<0.01	0.79	0.211
Hen housed production, %	86.06	85.75		85.65		0.09	86.54^a^	85.10^b^	<0.01	0.91	0.290
Unsaleable eggs^2^, %	0.90	0.71		0.81		0.51	1.23^a^	0.38^b^	<0.01	0.45	0.070
Double yolk eggs, %	0.47	0.43		0.42		0.34	0.57^a^	0.31^b^	<0.01	0.34	0.036
Soft shell eggs, %	0.20	0.15		0.17		0.46	0.26^a^	0.09^b^	<0.01	0.69	0.016
Cracked eggs, %	0.35	0.25		0.26		0.42	0.43^a^	0.14^b^	<0.01	0.99	0.034
Broken eggs, %	0.12	0.15		0.19		0.71	0.27^a^	0.04^b^	<0.01	0.69	0.024
Abnormal eggs, %	0.23	0.17		0.18		0.89	0.28^a^	0.11^b^	<0.01	0.33	0.029
Egg weight, g	59.44	59.54		59.54		0.70	58.84^b^	60.17^a^	<0.01	0.98	0.095
Specific gravity, g cm^−3^	1.091	1.091		1.091		0.50	1.0912^b^	1.0913^a^	<0.01	0.66	0.0001
Albumen height, Haugh unit	113.82	114.06		113.91		0.34	112.92^b^	114.94^a^	<0.01	0.16	0.289
Shell thickness, mm	0.371	0.371		0.370		0.71	0.370	0.371	0.40	0.92	0.0009

a,b Means within a main effect with different superscripts are significantly different (*P* ≤ 0.05).

1 SEM : standard error of the mean.

2 Unsaleable category = soft shell + cracked + broken + abnormal eggs.

**Table 8:** Effect of light-flicker frequency (F; 30, 90, or 250 Hz) during the pullet rearing phase on causes of mortality of 2 strains (S; Lohmann Brown-Lite [LB] and Lohmann LSL-Lite [LW]) of hens housed in conventional cages from 17 to 48 wk of age.

Mortality (%)	F			*P*-value	S		*P*-value	F x S *P*-value	SEM^1^
	30	90	250		LB	LW			
Total	1.04	1.13	1.74	0.29	2.20^a^	0.41^b^	<0.01	0.34	0.169
Cannibalism	0.26	0.35	0.69	0.19	0.84^a^	0.03^b^	<0.01	0.46	0.099
Metabolic	0.04	0.09	0.04	0.82	0.12^a^	0.00^b^	0.05	0.82	0.029
Infectious	0.09	0.17	0.22	0.53	0.26^a^	0.06^b^	0.04	0.18	0.048
Skeletal	0.00	0.04	0.00	0.40	0.03	0.00	0.34	0.40	0.014
Mechanical	0.26	0.22	0.22	0.90	0.41^a^	0.06^b^	<0.01	0.20	0.057
Other^2^	0.17	0.22	0.48	0.17	0.38	0.20	0.23	0.46	0.067
Unknown	0.22	0.04	0.09	0.17	0.17	0.06	0.14	0.36	0.041

a,b Means within a main effect with different superscripts are significantly different (*P* ≤ 0.05).

1 SEM: standard error of the mean.

2 Other category included diagnoses of: vent prolapse, internal ovulation, torsion of proximal magnum, focal rectal mucosal hemorrhage, trauma, egg bound, and blood clots in thorax.

## DISCUSSION

It was hypothesized that the visible F (30 and 90 Hz) would reduce pullet BW by reducing feed disappearance, due to increased stress and altered behavior due to the flicker. In line with the expectations of the Lohmann Tierzucht guidelines (2018), LB were heavier than LW, however, within each S, F did not impact BW. Previous research on F suggests that SP-789 laying hen BW is increased under lower frequencies (120 Hz vs. higher frequencies; Kavtarashvili and Gladin, 2022). The differences between the previous study and the present one may be due to the S and ages examined as well as the different range of F tested. However, similar to the present study, Raabe et al. (2023) found no effect of F on turkey tom BW. The previous studies did not examine F below 100 Hz which is on the cusp of what research states to be perceptible to hens, therefore, it is difficult to directly compare results (Nuboer et al., 1992; Jarvis et al., 2002; Railton et al., 2009; Lisney et al., 2012).

The term “feed disappearance” has been used because for the pullet phase, as pullets were observed dustbathing in the feeders and kicking feed out creating an unknown amount of feed wastage making it difficult to assess how much feed was being consumed. Feed disappearance has been defined as both consumed and wasted feed. It was hypothesized that F would reduce feed disappearance due to increased stress and potential behavioral changes, However, our stress indicators, heterophil-to-lymphocyte ratio and fear response (novel object test and tonic immobility) were unaffected by treatment up to 16 wk of age (McPhee, 2023). Strain dependent effects of F were noted during the 8 to 16 wk period indicating that LB reared under 30 Hz had a higher feed disappearance than other treatments, but this result was not reflected in the BW of LB reared under 30 Hz. However, it was found that 30 Hz increased the percentage of time pullets spent performing nutritive behaviors, described as percentage of time at the feeder and at the drinker (McPhee, 2023). This could suggest that pullets spent a higher percentage of time at the feeder and drinker, but that this time was spent wasting the feed as well as consuming it. Kavtarashvili and Gladin (2022) reported higher feed consumption in SP-789 hens housed under F of 120 Hz compared to higher F. Their results in combination with those of the present study may suggest that lowering F increases feed consumption and wastage, although Raabe et al. (2023) found no impact of F on feed consumption in turkey toms. It should be noted that Raabe et al. (2023) examined frequencies higher (165 Hz, 500 Hz, and 16 kHz) than those examined in the present or Kavtarashvili and Gladin (2022) studies.

It was hypothesized that the visible F treatments (30 and 90 Hz) would increase mortality, due to increased stress caused by the abnormal lighting system. It was found that the 30 Hz treatment resulted in more diagnoses in the “other” category, which included prolapse, choristoma, urates in ureter, and undefined. However, these “other” diagnoses have no clear common denominator, so it is unclear why there was an increase in the 30 Hz treatment. It should be noted that the incidence was also very low and poses little biological relevance. Strain dependent effects of F were noted for overall mortality where LW reared under 30 and 250 Hz had higher overall mortality than LB reared under the same treatments, however it should be noted that overall mortality for all treatments and S fell within the expected mortality range of 2 to 3% for these S (Lohmann Tierzucht, 2018). Mortality due to dehydration also demonstrated S dependent effects of F, but this was also a low-incidence diagnosis (0.60% in LW reared under 250 Hz) and does not likely indicate biological importance. Previous research conducted by Kavtarashvili and Gladin (2022) found a higher mortality due to aggression and cannibalism in SP-789 hens reared under 120 Hz compared to higher F, however, the present study found no mortalities due to aggression or cannibalism during the pullet phase. Potentially, if the flicker treatment had been extended into the laying period, differences in aggression may have arisen. Additionally, Raabe et al. (2023) found no differences in rates of injured or deceased turkey toms under different F. It is unclear how F may impact mortality, especially due to cannibalism and aggression, however, there were minimal impacts of F on mortality, and mortality due to cannibalism, in the S of pullets tested in the present study.

In terms of S differences, LW were heavier at 0 wk, likely influenced by breeder flock age, as the LW parent flock was older for the second trial. The LW pullets were more uniform than LB at 16 wk, which is to be expected and is consistent with previous research (Chew et al., 2021). Feed disappearance was higher for LB pullets during the 0 to 8 and 0 to 16 wk periods, as expected for LB (Lohmann Tierzucht, 2018). The LW pullets had higher incidences of yolk sac infections, a difference previously noted by Chew et al. (2021). However, less than 1% of chicks died due to yolk sac infection, which is less than the expected mortality for Lohmann chicks (Lohmann Tierzucht, 2018) and comparable to the values reported by Chew et al. (2021). Yolk sac infection is a common cause of first week mortality, so it is unsurprising that it was one of the leading causes of mortality in the present study (Olsen et al., 2012).

There were only minor carry-over effects of light flicker exposure during the rearing phase on hens in the production phase. The current study found F exposure during rearing resulted in small differences in LB hen BW and differences in hen day egg production, with no effect seen on feed efficiency or mortality. To our knowledge, no previous studies have evaluated the effects of light flicker exposure during the rearing phase and only two studies have evaluated the effects of light flicker during the egg production phase. While Widowski and Duncan (1996) did not evaluate performance, Kavtarashvili and Gladin (2022) have suggested that flicker exposure during the hen phase may influence hen performance.

While the birds in the current study appeared relatively unaffected by flicker, it should be noted that staff members reported the flicker to be aversive throughout the treatment period. Light flicker is detrimental to human health and effects include: headaches, eye strain, and anxiety (Wilkins et al., 2010) and therefore limiting the flicker present in poultry barns may be beneficial for producers.

## CONCLUSIONS

In conclusion, few effects of F were noted and in some cases responses were influenced by S. Future research could examine the responses of different commercially available S to F, in addition F treatment could be carried into the laying period to further examine the effects on productivity and mortality due to aggression.
